# Detection of collagen triple helix repeat containing-1 and nuclear factor (erythroid-derived 2)-like 3 in colorectal cancer

**DOI:** 10.1186/1472-6890-12-2

**Published:** 2012-02-09

**Authors:** Marco Palma, Lissett Lopez, Margarita García, Nuria de Roja, Tamara Ruiz, Julita García, Elisabet Rosell, Carmen Vela, Paloma Rueda, María-Jose Rodriguez

**Affiliations:** 1 Inmunología y Genética aplicada, S.A., Madrid, Spain; 2 Oryzon Genomics, S.A., Barcelona, Spain; 3 Inmunología y Genética Aplicada, SA, Calle Hermanos García Noblejas, 39 - 28037 Madrid, Spain

**Keywords:** Colorectal cancer, CRC, Cancer, Cancer biomarker, Biomarker, Triple Helix Repeat Containing-1, Nuclear factor (erythroid-derived 2)-like 3, CTHRC1, NFE2L3, Double antibody sandwich enzyme-linked immunosorbent assay, DAS-ELISA, DELFIA assay

## Abstract

**Background:**

Collagen Triple Helix Repeat Containing-1 (CTHRC1) and Nuclear factor (erythroid-derived 2)-like 3 (NFE2L3) may be useful biomarker candidates for the diagnosis of colorectal cancer (CRC) since they have shown an increase messenger RNA transcripts (mRNA) expression level in adenomas and colorectal tumours when compared to normal tissues.

**Methods:**

To evaluate CTHRC1 and NFE2L3 as cancer biomarkers, it was generated and characterised several novel specific polyclonal antibodies (PAb), monoclonal antibodies (MAbs) and soluble Fab fragments (sFabs) against recombinant CTHRC1 and NFE2L3 proteins, which were obtained from different sources, including a human antibody library and immunised animals. The antibodies and Fab fragments were tested for recognition of native CTHRC1 and NFE2L3 proteins by immunoblotting analysis and enzyme-linked immunosorbent assay (ELISA) in colorectal cell lines derived from tumour and cancer tissues.

**Results:**

Both, antibodies and a Fab fragment showed high specificity since they recognised only their corresponding recombinant antigens, but not a panel of different unrelated- and related proteins.

In Western blot analysis of CTHRC1, a monoclonal antibody designated CH21D7 was able to detect a band of the apparent molecular weight of a full-length CTHRC1 in the human colon adenocarcinoma cell line HT29. This result was confirmed by a double antibody sandwich enzyme-linked immunosorbent assay (DAS-ELISA) with the monoclonal antibodies CH21D7 and CH24G2, detecting CTHRC1 in HT29 and in the colon adenocarcinoma cell line SW620.

Similar experiments were performed with PAb, MAbs, and sFab against NFE2L3. The immunoblot analysis showed that the monoclonal antibody 41HF8 recognised NFE2L3 in HT29, and leukocytes. These results were verified by DAS-ELISA assay using the pairs PAb/sFab E5 and MAb 41HF8/sFab E5.

Furthermore, an immunoassay for simultaneous detection of the two cancer biomarkers was developed using a Dissociation-Enhanced Lanthanide Fluorescent Immunoassay technology (DELFIA).

**Conclusions:**

In conclusion, the antibodies obtained in this study are specific for CTHRC1 and NFE2L3 since they do not cross-react with unrelated- and related proteins and are useful for specific measurement of native CTHRC1 and NFE2L3 proteins. The antibodies and immunoassays may be useful for the analysis of CTHRC1 and NFE2L3 in clinical samples and for screening of therapeutic compounds in CRC.

## Background

Colorectal cancer (CRC) is one of the most common causes of cancer-related death in United States and Western World [1]. During 2008, it was reported more than 140000 new cases of CRC with more than 49000 deaths related to it [2]. Many patients with CRC may develop liver metastases at some point during the course of their disease [3]. Generally, colorectal cancer does not show any symptoms until advanced stages of the disease. Therefore, early diagnosis is very important to identify adenomatous tissue in pretumoral lesions to remove it before it becomes neoplastic tissue.

Many molecular markers, including the carcinoembryonic antigen (CEA), have been exploited for detecting CRC. However, these biomarkers are not providing sufficient sensitivity and reliability for the early detection of CRC [4-8]. Thus, there is an urgent demand for research into novel molecular markers that can serve as diagnostic and prognostic markers for CRC. Collagen Triple Helix Repeat Containing-1 (CTHRC1) and Nuclear factor (erythroid-derived 2)-like 3 (NFE2L3) may be useful biomarker candidates for the diagnosis of CRC since they have shown an increase messenger RNA transcripts (mRNA) expression level in colorectal tumours when compared with normal tissues [9-12].

CTHRC1 is a 30 kDa secreted protein that has the ability to inhibit collagen matrix synthesis. It is highly expressed in cartilage, developing bones, and myofibroblasts during skin wound healing and its expression is increased in fibroblasts and chondrocytic cells in response to TGF-beta family members [13]. CTHRC1 inhibit TGF-beta signalling which can lead to a reduction in collagen type I deposition during vascular remodelling. In addition, it has been shown that CTHRC1 is over-expressed in colorectal cancer [9-11]. In addition, CTHRC1 is implicated in breast cancer, suggesting that CTHRC1 expression in this type of tumour is associated with cancer tissue invasion and metastasis [14]. In recent studies it was suggested that CTHRC1 inhibition may represent a potential method for decreasing melanoma resistance to conventional chemotherapy [15].

NFE2L3 is the third member of the family of NF-E2-related- factors which are well-known for their participation in the basal expression and induction of defensive genes in response to antioxidants [16]. NFE2L3 has been described as a regulator of the antioxidant-response element (ARE) [17]. Also, recently, a high mRNA NFE2L3 expression has been detected in human placenta and it has been related to placental and developmental expression [18]. NFE2L3 was also expressed in relation to ovarian serous papillary carcinoma [19], solid tumour formation in stem cells [20], and testicular seminoma diagnosis [21]. NFE2L3 is up-regulated in colorectal cancer [12]. Recent studies showed the first in vivo function of NFE2L3 and its link to tumour development using a NFE2L3 - deficient mice. This study showed that the absence of NFE2L3 predisposes mice to lymphoma development, suggesting a protective role of this transcription factor in hematopoietic malignancies [22].

In this study, it had been obtained specific polyclonal antibodies (PAbs), monoclonal antibodies (MAbs), and soluble Fab fragments (sFAbs) against CTHRC1 and NFE2L3 and some immunological assays for their detection in biological samples were established.

## Methods

### Cell culture and total cell extract preparation

The human colon adenocarcinoma cell lines HT29 and SW620 were kindly supplied by Oryzon Genomics (Barcelona, Spain), originally acquired from the American Tissue Culture Collection (ATCC)(Manassas, VA, USA).

The HT29 and SW620 cell lines were cultured with F12 medium supplemented with 10% heat-inactivated fetal bovine serum (FBS) (Gibco, Grand Island, NY). The cells were grown to confluency at 37°C and 5% CO_2_. To obtain total cell extracts, cell monolayers were washed twice with cold phosphate buffered saline (PBS), and then were scraped and centrifuged at 1500 × *rpm *for 5 min at 4°C. The cellular pellets were resuspended in lysis buffer (10 mM Tris-HCl, pH 7.5, 140 mM NaCl, 5 mM EDTA, and 1.5% v/v Triton X-100) supplemented with protease inhibitor cocktail complete EDTA free (Roche, Basilea) and sonicated.

### Preparation of bovine leukocytes suspensions

A blood sample from cow was collected for leukocytes isolation. The cow received humane care in accordance with the Guidelines for the accommodation and care of animals used for experimental and other scientific purpose according Animal Wellfare Laws (National Laws RD 1201/2005). The animal experiment was approved by Ethical Committee "Nucleo Zoosanitario" (No ES-80790000095). The sample was collected into heparin (Sigma, Inc., St.Louis, MO) and centrifuged at 1000 × *g *for 15 min at room temperature (RT). The supernatant was removed and the pellet was treated with lysis buffer (310 mM NH_4_Cl, 24 mM NaHCO_3_, 0.5 mM Na_2 _EDTA, pH 7.4) for 15 min at room temperature. The sample was centrifuged again at 1000 × *g *during 15 min and the supernatant was removed. The pellet was resuspended in 400 μl 25 mM bicarbonate buffer.

### Human lymphocytes purification

Fresh human lymphocytes were isolated from 50 ml blood from a healthy volunteer blood donor by using Histoplaque density gradients purchased from Sigma (St.Louis, MO), following the protocol of the manufacturer. The human blood sample was obtained in accordance with the declaration of Helsinki from 1997 and its revision from 2004 and Spanish Biomedical Law 121/000104. Written informed consent was obtained from the participants after oral and written information was provided. The local ethics committee and the ethic committee of the consortium Oncnosis Pharma AIE approved all procedures. The lymphocytes were lysed by lysis buffer (10 mM Tris-HCl, pH 7.5, 140 mM NaCl, 5 mM EDTA, and 1.5% v/v Triton X-100) supplemented with protease inhibitor cocktail complete EDTA free (Roche, Basilea) and sonicated.

### Cloning, expression and purification of recombinant CTHRC1 and NFE2L3

A full-length of the human *CTHRC1 *gene was cloned into the Gateway System expression vector pDEST17 (Invitrogen, Carlsbad, CA) from pDONR233 (Open Biosystem BC014245), by recombination based on the lambda recombination system. The sequence was confirmed with an automatic DNA sequencer. The new vector was called pDEST17-*CTHRC1*.

A truncated form of *NFE2L3 *gene (844nt-2345nt)(corresponding to residues 30-694) was amplified by PCR using cDNA obtained by reverse transcription of total RNA from tumour cell line SW620 as described previously [23]. Two oligonucleotides NFE2L3 REVERSE (5'-CTCACTTTCTCTTTCCCTTTTGGG-3') and NFE2L3 FORWARD (5'-AGAGAAAAGCACGAAGCTGTG-3') were designed and the amplification reaction was carried out in a total volume of 50 μl with 1 μl cDNA, 1 U DNA Taq polymerase (Roche), 200 μmol of each deoxyribonucleotide triphosphate, and 100 ng of each primer. Amplification involved 35 cycles at 94°C during 30 seconds, 55°C 30 seconds, 72°C 2 minutes 35 cycles, and a final extension step at 72°C during 10 minutes. The PCR product was purified and cloned into the TOPO Gateway vector (Invitrogen, San Diego, CA). The cloned fragment was subsequently transferred to the expression vector pDEST17 by using the Gateway system (Invitrogen). The new vector was called pDEST17-*NFE2L3*. All sequences were confirmed by sequencing.

In both cases the genes concerned were expressed as a fusion protein that included His-Tag. The recombinant CTHRC1 and NFE2L3 proteins were expressed by transformation of *E.coli *BL21 strain (Invitrogen) cells. Overnight cultures of transformed bacteria in LB supplemented with 100 μg/ml of ampicillin and 50 μg/ml carbenicillin were inoculated in fresh medium. Cultures were grown at 37°C until they reached mid-log phase (OD600 = 0.5). At that moment, expression of the CTHRC1 and NFE2L3 recombinant proteins were induced with the addition of L-arabinose to a final concentration of 0.2% w/v and incubated during three hours at 37°C. Then, cultures were centrifuged, resuspended in lysis buffer containing 400 mM NaCl, 100 mM KCl, 10% glycerol, 0.5% Triton X-100, and 10 mM Imidazol. Due to the insolubility of the proteins, an additional step was done consisting in a ClGn-6 M extraction and finally an additional purification step in a column prepacked with Ni Sepharose (His GraviTrap™ from GE Healthcare, Buckinghamshire, UK) was carried out.

### Production of anti-NFE2L3 polyclonal antibodies

Purified recombinant NFE2L3 protein was used to immunize rabbits. All rabbits received humane care in accordance with the Guidelines for the accommodation and care of animals used for experimental and other scientific purpose according Animal Wellfare Laws (National Laws RD 1201/2005). The animal experiment was approved by Ethical Committee "Nucleo Zoosanitario" (No ES-80790000095). Aliquots containing 200 μg of protein in 500 μl of PBS were mixed with an equal volume of Freund's complete adjuvant for the first injection and Freund's incomplete adjuvant for boosters. All injections in rabbits were administered subcutaneously in the leg of the rabbit. Injections were repeated 3 times at 2-week intervals. Immunoglobulins presented in serum were purified by a protein G column. The protocol suggested by the manufacturer was followed (GE Healthcare Live Science, Uppsala, Sweden). The purified immunoglobulins were dialysed against PBS, diluted with glycerol (1:1) and stored at -20°C.

### Production of anti-CTHRC1 and NFE2L3 monoclonal antibodies

After immunization of mice with CTHRC1 or NFE2L3, several MAbs specific for CTHRC1 or NFE2L3 were obtained. Immunisation protocols, production and purification of MAbs were performed as previously described [24,25]. All mice received humane care in accordance with the Guidelines for the accommodation and care of animals used for experimental and other scientific purpose according Animal Wellfare Laws (National Laws RD 1201/2005). The animal experiment was approved by Ethical Committee "Nucleo Zoosanitario" (No ES-80790000095).

### Production of anti-NFE2L3 Fab fragments

The antibody library FAB-310 (Dyax Corp, Cambridge, MA) was used to select recombinant monoclonal antibodies in the form of Fab fragments using the phage display technology. FAB-310 is a human Fab library that has an unique combination of immunoglobulin sequences captured from human donors and synthetic diversity in key antigen contact sites in heavy-chain complementarity-determining regions 1 and 2 [26]. The Fab fragments in the FAB-310 library are expressed on the surfaces of M13 fused with pIII protein. Fab-310 has a diversity of 3.5 × 10^10 ^(1.5 × 10^10 ^of kappa and 2 × 10^10 ^of lambda). The selection procedure was done as it was recommended by the manufacturer in the experimental handbook of the product (Dyax Corp, Cambridge, MA).

Positive Fab clones against recombinant CTHRC1 and NFE2L3 were reformated to soluble Fab fragments (sFab) by removing the pIII gene. The plasmids pMID21 carrying the Fab fragments were digested with *Mlu*I enzyme, re-ligated and transformed into *E. coli *TG-1 starin. 94 randomly chosen individual colonies from each selection round were analysed as recommended by the manufacturer (Dyax Corp, Cambridge, MA). Supernatant material was analysed in indirect ELISA using 0.1 μg CTHRC1, NFE2L3 or a non related biomarker as negative control protein per well and detecting bonded sFabs with anti C-myc HRP (Pierce, Waltham, MA) or anti Fab peroxidase-conjugated antibodies (Sigma). Specific fragments were defined as sFabs clones that showed a sign of ELISA at least 3 times higher in the plate covered with CTHRC1 or NFE2L3 than in the plate covered with negative control.

sFab producing clones were grown until they reached a cellular density of 0.9 (OD600) on a stirrer at 37°C. sFab production was induced with a final Isopropyl-beta-d- thiogalactopyranoside (IPTG) concentration of 1 mM and the cultures were grown on a stirrer at 30°C during the night. sFabs were separated from bacterial sediment through centrifugation and purified by affinity with protein A-Sepharose (GE Healthcare, Buckinghamshire, UK).

### Antibodies characterization

To determine the binding specificity of PAbs, MAbs, and sFab, indirect ELISA trials were carried out using non conjugated or biotin-conjugated antibodies. CTHRC1 or NFE2L3 (30-694) were used to cover 96-well Maxisorp plates (Nunc) in a 1-5 μg/ml concentration. The bindings with increasing biotin-conjugated antibodies concentrations were measured with streptavidin-peroxidase (Sigma, Inc., St.Louis, MO). PAb, MAbs, or sFabs binding with other non-related proteins, such as lysozyme, fibrinogen, albumin, ovalbumin, human IgG purchased from Sigma (St. Louis, MO) or with other non-related cancer biomarkers were also determined.

### Western blotting

Immunologic detection of proteins was performed by Western blot. 50-100 μg cellular extract was loaded in a sodium dodecyl sulfate-polyacrylamide gel (SDS-PAGE) and then transferred to a nitrocellulose membrane. The MAb CH21D7 against CTHRC1 was used in a concentration of 25 μg/ml. The peroxidase-conjugated PAb against NFE2L3 was used in a dilution of 1:2500. The monoclonal antibody 41HF8 and the sFab E5 against NFE2L3 were used in a concentration of 10 μg/ml. All secondary antibodies coupled to horseradish peroxidase directed against mouse IgG (Amersham) and rabbit IgG were diluted 1:5000 and anti human Fab fragment 1:2000 (Sigma, Inc., St.Louis, MO).

### Peroxidase and biotin conjugation of antibodies

The PAb were conjugated with peroxidase using the periodate coupling method described by Nakane and co-workers [27]. The PAbs, MAbs and sFabs were biotinylated using (+)-Biotin N-hydroxysuccinimide ester following the protocol developed by Bayer and Wilchek [28].

### Colorimetric DAS-ELISA

Three assay formats were developed: one format to detect CTHRC1 and two formats to detect NFE2L3.

In the CTHRC1 DAS-ELISA, the MAb CH21D7 was used as captured antibody with a concentration of 0.5 μg/ml and biotin-conjugated CH24G2 as detection antibody with a concentration 0.5 ng/ml.

In the NFE2L3 DAS-ELISA, the sFab E5 was used as captured antibody with a concentration of 1.2 μg/ml and the PAb or biotin-conjugated 41HF8 as detection antibody with a concentration of 5 ng/ml. In an alternative DAS-ELISA was used PAb as captured antibody with a concentration of 5 ug/ml and biotin-conjugated 41HF8 as detection antibody with a concentration of 5 ng/ml.

The plates were blocked with 3% BSA in PBS 0.1% Tween. Cellular extracts, or control antigens were added to the wells and incubated 1 h 24°C or overnight at 4°C. The control antigens were recombinant proteins (CTHRC1 or NFE2L3). The negative control was NFE2L3 in the CTHRC1 DAS-ELISA and CTHRC1 in the NFE2L3 DAS ELISA. All further incubations were performed at room temperature. The biotin-conjugated antibodies were detected with the streptavidin-peroxidase (Sigma, Inc., St.Louis, MO).

### Dissociation-enhanced lanthanide fluorescent immunoassay

The colorimetric DAS-ELISAs against CTHRC1 and NFE2L3 were converted to a single Dissociation-Enhanced Lanthanide Fluorescent Immunoassay (DELFIA). Chessboard reagent titration experiments were carried out to define optimal assay parameters. In the DELFIA assay, sFab E5 and MAb CH21D7 were used as captured antibodies with a concentration of 0.5 and 0.06 μg/well respectively in yellow plates. The recommendations of the manufacturer were followed (Perkin Elmer, Turku, Finland). The plates were blocked with blocking solution (50 mM NaH_2_PO_4_, 6% trehalose, 0.1% BSA-TSA, 0.1% Germal II)(Sigma, Inc., St.Louis, MO). Recombinant CTHRC1, NFE2L3 or a non-related cancer biomarker as negative control protein were added to the wells and incubated at room temperature during 1 hour. The captured antigens were detected with a mixture of PAb against NFE2L3 and biotin-conjugated CH24D7, followed by a mixture of Europium-labelled anti rabbit antibody and Samarium-labelled streptavidin. The signal was enhanced adding enhancement solution (Perkin Elmer, Turku, Finland). The fluorescence was measured with Envision Multilabel Plate reader (Perkin Elmer, Turku, Finland). Antibody levels were shown as fluorescence units.

## Results

### Characterization of the recombinant proteins and antibodies

Several antibodies including PAb, MAbs and Fab fragments were purified and characterized. They were originally identified by their recognition of CTHRC1 or NFE2L3 by indirect ELISA. The specificity of CH21D7 and NFE2L3 was validated by Western blots and DAS-ELISA. During the course of the screening those antibodies that did not present cross-reactivity with not related proteins as human fibrinogen, lysozyme, ovalbumin, bovine serum albumin (BSA) and human IgGs, not either with other cancer biomarkers (data not shown) or total *E. coli *cell extract were considered specific. Also, those antibodies that presented cross reactivity against the mentioned proteins were discarded. The sFabs against CTHRC1 were discarded since they did not recognize the recombinant protein in Western blot, as well some MAbs against NFE2L3. The MAbs CH21D7 and CH24G2 against CTHRC1, and the PAb, the MAb 41HF8, and the sFab E5 against NFE2L3 were considered specific for their antigens and were used in further analysis.

### Immunoblot analysis of cellular extracts

The obtained PAb, MAbs and sFab were tested for specificity on Western blots. The SDS-PAGE was loaded with cell extracts from two colon cancer cell lines (HT29 and SW620), leukocytes and lymphocytes and analyzed for NFE2L3 detection.

**Figure 1:**
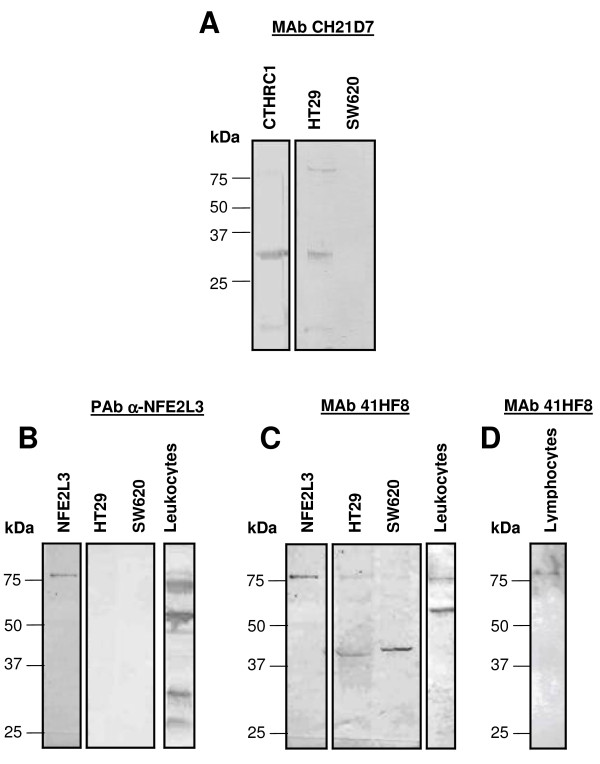
**Detection of CTHRC1 and NFE2L3 in Western Blot**. **A**: Recombinant- and native CTHRC1 analysed in total protein extracted from HT29, and SW620 with MAb CH21D7. **B, C & D**: Recombinant- and native NFE2L3 analysed with PAb anti-NFE2L3 (**B**) or MAb 41HF8 (**C**) in total protein extracted from HT29, SW620, leukocytes and lymphocytes (**D**)

The results indicated that the MAb CH21D7 displayed high specificity toward the native protein expressed in the cell line HT29, detecting a band of the apparent molecular weight of a full-length CTHRC1 (~28 kDa) (Figure 1A), however, non protein was detected in SW620 or in the negative control (*E.coli *protein extract). The MAb CH24G2 against CTHRC1 recognized only the recombinant and not the native CTHRC1 (data not shown).

The PAb anti NFE2L3 recognized the recombinant protein and several bands which had the molecular weight of 77-, 65-, 30- and 28 kDa (Figure 1B) only in leukocytes but not the cancer cell lines HT29 and SW620. The MAb 41HF8 detected two bands of 77-and 65 kDa (Figure 1C) in leukocytes and a 77 kDa band in lymphocytes (Figure 1D). This antibody detected two bands of the molecular size of 77- and 44 kDa in HT29 and SW620. The sFab E5 against NFE2L3 recognized only the recombinant and not the native NFE2L3 (data not shown). A summary of Western blot results is shown in Table 1.

**Table 1:** Summary of the analysis of CTHRC1 and NFE2L3 by Western blot

Biomarkers and antibodies	Cell extracts analyzed					
	
	HT29	SW620	*E.coli *cell extract	Lymphocytes	Leukocytes	Other
**CTHRC1 detection by**						
MAb CH21D7	**Yes**	No	No	ND	ND	ND
MAb CH24G2	No	No	No	ND	ND	ND

**NFE2L3 detection by**						
PAb α-NFE2L3	No	No	No	ND	**Yes**	ND
MAb 41HF8	**Yes**	**Yes**	No	**Yes**	**Yes**	ND
sFab E5	No	No	No	ND	ND	ND

ND: not determined

### Development of DAS- ELISA

**Figure 2:**
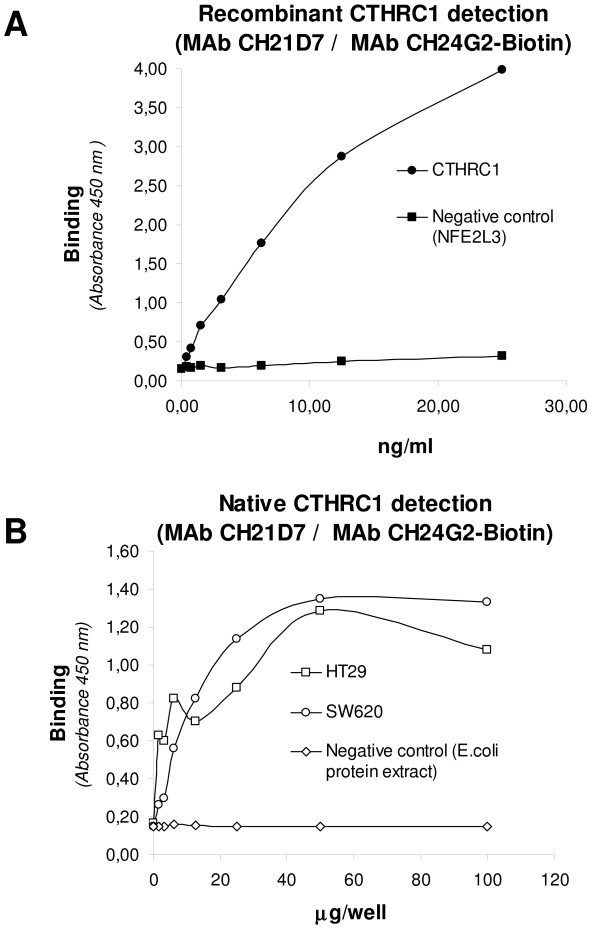
**CTHRC1 detection by DAS-ELISA**. **A**: Detection of the recombinant CTHRC1- and negative control protein (NFE2L3). **B**: Detection of the native CTHRC1 protein in colon cell lines HT29 and SW620, and *E. coli *protein extract used as negative control. The MAb CH21D7 was used as captured antibody and biotin-conjugated CH24G2 as detection antibody. The cellular extracts or control antigens were incubated in the wells during 1 h at 24°C or overnight at 4°C.

The MAbs CH21D7 and CH24G7 were evaluated as antibody pair in a sandwich ELISA for CTHRC1 detection, resulting in CH21D7 as capture antibody, and biotin-conjugated CH24G7 as the detection antibody (Figure 2A). The chessboard reagent titration showed that optimal concentration for the assay was 0.5 μg/well of CH21D7 as capture antibody and dilution 1:16000 of biotin-conjugated CH24G7 as detection antibody. The assay sensitivity, which was calculated by comparing the signal of the CTHRC1 detection with the signal giving by the negative control protein, was 2.0 ng/ml.

The PAb, MAb 41HF8 and sFab E5 specific for NFE2L3 were combined to find the best antibody pairs in sandwich ELISA for NFE2L3 detection.

**Figure 3:**
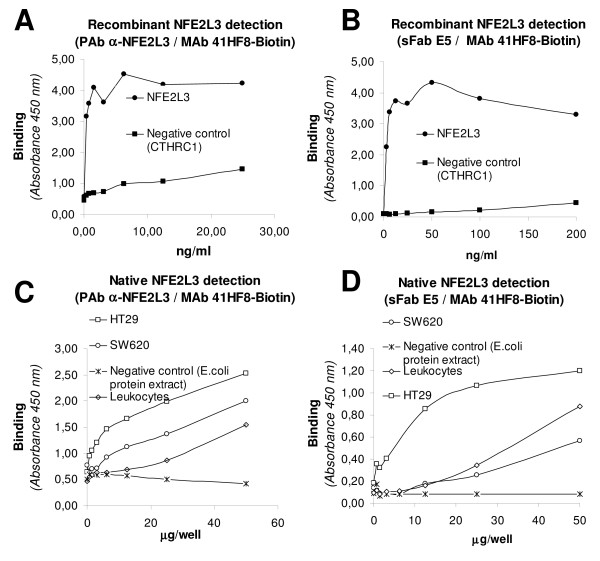
**NFE2L3 detection by DAS-ELISA**. **A & B: **Detection of the recombinant NFE2L3- and negative control protein (CTHRC1) and analysis of NFE2L3 in protein extracts from HT29, SW620, leukocytes, and *E. coli *protein extract with the antibodies pair PAb α-NFE2L3 and MAb 41HF8. **C & D: **Detection of the recombinant NFE2L3- and negative control protein (CTHRC1) and analysis of NFE2L3 in protein extracts from HT29, SW620, leukocytes, and *E. coli *protein extract with the pair sFab E5 and MAb 41HF8. The samples were incubated during 1 h at 24°C or overnight at 4°C.

The PAb (Figure 3A and 3B) or sFab E5 (Figure 3C and 3D) were used as capture antibody, and biotin-conjugated PAb or biotin-conjugated 41HF8 or biotin-conjugated sFab E5 as detection antibodies.

The assay sensitivity of the NFE2L3 DAS-ELISA was calculated by comparing the signal of the NFE2L3 detection with the signal giving by the negative control proteins. The assays sensitivities were 0.5-2.0 ng/ml.

### DAS-ELISA analysis of cellular extracts

In an initial attempt to evaluate CTHRC1 and NFE2L3 as biomarkers for cancer and the obtained antibodies for their identification, the DAS- ELISA was used to measure CTHRC1 and NFE2L3 in colon cancer cells using known colon cancer cell lines as a model. The CTHRC1 DAS-ELISA clearly detected the antigen in the cell line HT29 and SW620, however, not signal was obtained in the *E.coli *protein extract used as negative control (Figure 2B). The NFE2L3 DAS-ELISA clearly detected the antigen in the cell line HT29 and with a lightly signal in the cell line SW620 and in leukocytes (Figure 3B and 3D). Non signal was detected in the *E.coli *protein extract which was used as negative control.

### Multiplex ELISA development

**Figure 4:**
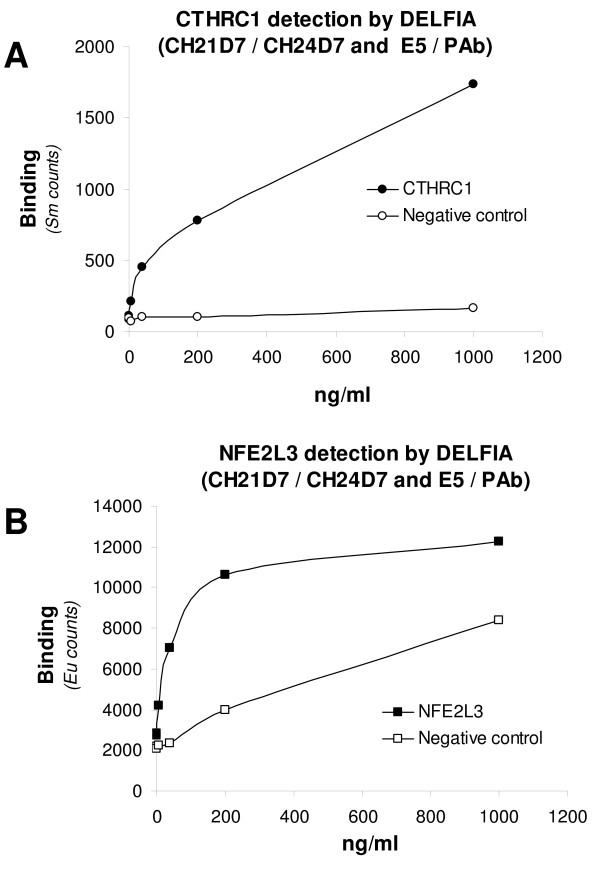
**Double detection of CTHRC1 and NFE2L3 by DELFIA**. **A: **Detection of recombinant CTHRC1 and a non-related biomarker as negative control protein. **B: **Detection of recombinant NFE2L3 and a non-related biomarker as negative control protein. The sFab E5 and MAb CH21D7 were immobilised in DELFIA yellow plate. The plates were blocked with blocking solution. A mixture of recombinant CTHRC1 and NFE2L3 was added to the wells. The captured antigens were detected with rabbit PAb α-NFE2L3 and biotin-conjugated CH24D7, followed by Europium-labelled anti rabbit antibodies and Samarium-labelled streptavidin. The signal was amplified by enhancement solution. The amount of antibodies was detected by dissociation-enhanced time-resolved fluoroimmunoassays research fluorometer.

The two colorimetric DAS-ELISAs developed were converted to one DELFIA assay to multiplex the CTHRC1 and NFE2L3 detection (Figure 4). DELFIA technology can be useful for the CTHRC1 and NFE2L3 detection because it offers high sensitivity, wide dynamic range, stability, possibility of multiplexing, and reducing the amount of samples. The antibodies pairs selected for the assay were chosen to avoid as much background as possible. Chessboard reagent titration experiments were carried out to define optimal assay parameters. The assay was optimised as it was recommended by the Perkin Elmer DELFIA guide. The combination of antibodies pair chosen for the assay were MAbs CH21D7/CH24D7 for CTHRC1 detection and sFab E5/PAb for NFE2L3 detection. The chessboard reagent titrations showed that the optimal concentrations of antibodies were 0.06 μg/well MAb of CH21D7 and 0.5 μg/well of E5 as capture antibodies and a dilution 1:40000 of PAb and 1:500 of CH24D7 as detection antibodies. Washing condition was optimized to 6 washes after incubation with samples and detection antibodies to reduce background. The shaking speed was optimised to 125 rpm and the incubation time with enhancement solution to 15 min to increase the signal. The CTHRC1 readings were not affected by the presence of the anti-NFE2L3 reagents or NFE2L3 reading by the presence of anti-CTHRC1 reagents. The sensitivities of the assays for CH21D7 and NFE2L3 detection were 0.5 - 2.0 ng/ml.

## Discussion

The effort of this study was focus on CTHRC1 and NFE2L3 as biomarkers stimulated by the strong evidences of the differential expression of CTHRC1 and NFE2L3 in CRC. Already in 2006 [29], it was demonstrated with the patent EP2177628A2 that the CTHRC1 was related to CRC and could be used as marker for its diagnosis since CTHRC1 was over-expressed in CRC reaching mRNA level of 22-fold compared to normal cells. In another study, it was shown that the CTHRC1 expression was dramatically up-regulated in several cancer types including CRC [30]. In the same way, the relation of NFE2L3 to cancer was studied by other investigators, showing that 20 genes, including the gene encoding NFE2L3, were up-regulated in colorectal cancer cells [12] and in adenomas when compared with normal tissues [9-12]. Based on these investigations, it is clear that the information available indicates that CTHRC1 and NFE2L3 proteins are useful candidates to determine the progress of CRC. For example, in our patent EP2008/010665, we described a method for the diagnosis and/or prognosis of colorectal tumour by NFE2L3 detection [31].

Most of the studies of the CTHRC1 and NFE2L3 expression have been focused mainly on the analysis of the mRNA levels, existing almost not results about their protein levels. Therefore, it is really important to have specific antibodies against these two biomarkers to facilitate further studies and make possible to set up immunological assays for CRC diagnosis. Therefore, the purpose of the present study was to select PAb, MAbs, and sFab fragments against CTHRC1 and NFE2L3 proteins and to develop immunoassays for CRC diagnosis. We obtained several specific antibodies against the two biomarkers which were analyzed carefully. However, we concentrated our efforts on analysing CTHRC1 and NFE2L3 in cell lines since they are the most frequently used living systems in research and they can be used as model for biomarker detection [32-34]. In addition, leukocytes and lymphocytes were used for NFE2L3 detection since there is some information showing that NFE2L3 mRNA is highly expressed in B cell and monocyte lineage [16].

Our results indicated that the obtained antibodies recognized their corresponding native proteins produced in cell lines derived from cancer cells. Western blot showed a band of the apparent molecular weight of a full-length CTHRC1 in HT29 cells which corresponds to the theoretical size of 26.2 kDa according to Collagen triple helix repeat-containing protein 1 precursor from UniProtKB at http://www.uniprot.org/uniprot/Q96CG8[35]. The CTHRC1 detection in CRC cells was proved by a DAS-ELISA DAS with the MAbs CH21D7 and CH24G2. Also, we were able to detect with the assay the CTHRC1 protein in HT29 and SW620 cell lines, but not in the negative control protein extract. This DAS-ELISA had a sensitivity of 2.0 ng/ml. In the same way, we showed the NFE2L3 protein expression in CRC cells. Several protein bands were detected by Western blot in CRC cell lines, leukocytes and lymphocytes corresponding to the molecular weight of 44-, 65-, and 77 kDa. The protein band pattern obtained in our result was formed probably due the processing of the native protein. In theory, the expected molecular weight of NFE2L3 should be 76.1 kDa according to Nuclear factor erythroid 2-related factor 3 from UniProtKB at http://www.uniprot.org/uniprot/Q9Y4A8[36], however, in the practice the size of NFE2L3 can vary according to proteins glycosylation or proteolysis. There are some indications of multiple NFE2L3 formation shown mainly in the study of Zhang *et al. *Our results are partially in concordance with the NFE2L3 size shown in this study in which a synthesized protein of approximately 96-kDa was subsequently converted into isoforms of approximately 90, 80, and 70 kDa [37]. In addition of the bands mentioned above, their work showed two smaller bands, hypothesizing that they are created through either translation from two internal start codons at Met173 and Met211 or from the proteolytic cleavage occurring within a central region [37]. The native NFE2L3 detection was confirmed by DAS-ELISA with PAb, MAbs, and sFabs against NFE2L3 in CRC cells and leukocytes. Also, we were able to detect with this DAS-ELISA the NFE2L3 protein in HT29 and SW620 cell lines, but not in the negative control protein extract. The assays sensitivities were 0.5 - 5.0 ng/ml.

Multiplex detection of biomarkers can be useful in diagnosis of CRC, therefore, we optimized an immunoassay for duplex detection of CTHRC1 and NFE2L3. We were able to combine the antibodies pairs of two developed colorimetric DAS-ELISA to one DELFIA assay without disturbing each other in the measurement. When the two colorimetric DAS-ELISA were converted to DELFIA assays for single measurement, the sensitivity was increase to 3-fold. When two individual DELFIA assays were combined, the sensitivity decreased to range of the colorimetric DAS-ELISA. We were able to develop an assay to detect the two biomarkers simultaneously. The DELFIA assay was successfully developed. Even, when the sensitivity was not improved in this case with DELFIA several advantages have been incorporated to the original DAS-ELISA with this technology. Some of these advantages can be useful in medical diagnostic including cost-, time-, and samples- reduction and signal stability. The sensitivity of our DELFIA assay is in the process to be improved by replacing the pre-labelled secondary antibodies with primary lanthanide-labelled antibodies.

It will be interesting to test our antibodies and assays with other biological samples than cell lines such as serum or tissues from CRC patients. In a first effort to optimize the assay conditions for serum analysis with our assays, it was performed spike-in experiments with negative serum to determine if serum component can affect the detection level of CTHRC1 and NFE2L3. The diagnostic/prognostic in the serum of patients can be limited by the presence of highly abundant albumin and immunoglobulins that constitute approximately 60-97% of the total serum proteins [38] or by other components. However, in our study we were not able to see any differences between the detection with and without serum, indicating that measurement of our biomarkers is unaffected by highly abundant proteins in serum (data not shown). We are in the process to improve the assay sensitivity and performance by labelling the PAb, MAb CH24D7 and MAb 41HF8 directly with Samarium and Europium. An additional application of our DELFIA CTHRC1 NFE2L3 assay may be mass screening of therapeutic compounds relating to CRC.

## Conclusions

In conclusion, the present study offers a simple and reliable method for the diagnosis of CRC based on CTHRC1 and NFE2L3 detection by a double sandwich antibody ELISA or DELFIA technology. The selected antibodies can be useful for detection of the two biomarkers in tissue samples or serum and in studies of these two biomarkers.

## Abbreviations

pAb: Polyclonal antibody; mAb: Monoclonal antibody; sFabs: Soluble Fab fragments; CRC: Colorectal cancer; RT-PCR: Real time-polymerase chain reaction; PBS: Phosphate buffered saline; BSA: Bovine serum albumin; IPTG: Isopropyl-beta-d- thiogalactopyranoside; CDRs: Variable domain; VH: Variable domains of the heavy chain; VL: Variable domains of the light chain; DMSO: Dimethyl sulfoxide; BNHS: Biotinyl-N-hidroxysuccinimide ester; SDS-PAGE: Sodium dodecyl sulfate-polyacrylamide gel; ELISA: Enzyme-linked immunosorbent assay; DAS-ELISA: Double sandwich antibody ELISA; PBS: Phosphate buffered saline; PEG: Polyethylene glycol; TEA: Triethylamine; DMEM: Dulbecco's modified Eagle's médium; RT: Reverse transcription; PCR: Polymerase chain reaction; FBS: Fetal bovine serum.

## Competing interests

The authors declare that they have no competing interests. Oryzon Genomics, S.A and Inmunología y Genética declared that they have not competing interests. Contractual agreements were signed under the consortium Oncnosis Pharma, AIE, between involved companies and employees to avoid conflict of competing interests. The antibodies against NFE2L3 were protected by patent EP2008/010665. The publication of this study was approved by "Oncnosis Pharma AIE's committee."

## Authors' contributions

MP contributed to study design, data interpretation, carried out the experiments and preparation of the manuscript. LL and MG contributed to study design, experimental work and data interpretation and manuscript revision. NR, TR and JG contributed to perform part of the experimental work. CV and ER contributed with coordination of the entire study. MJR and PR coordinated the study design, data interpretation and manuscript revision. All authors have read and approved the final manuscript.

## Authors' information

ER currently holds a position at Lacareda network S.L., Enric Granados, 43, 08008 Barcelona, Spain. ER current email address is elisabet.rosell@lacareda.com.

## Pre-publication history

The pre-publication history for this paper can be accessed here:

http://www.biomedcentral.com/1472-6890/12/2/prepub
